# Lung transplantation for severe PAH – How far can we go? Pro – lung transplantation

**DOI:** 10.1016/j.jhlto.2026.100701

**Published:** 2026-10-06

**Authors:** K. Hoetzenecker, L. Savale

**Affiliations:** a Vanderbilt University Medical Center, Department of Thoracic Surgery, Nashville, TN; b Université Paris-Sud 11, Faculté de Médecine, Le Kremlin-Bicêtre, Clamart, France

**Keywords:** lung transplantation, pulmonary arterial hypertension, heart/lung transplantation, cardiac remodeling

## Introduction

Historically, heart-lung transplantation was established even before the first successful lung transplant. The pioneering work of Bruce Reitz and Norman Shumway paved the way for the technical feasibility of transplanting a heart-lung block. In the early days of thoracic transplantation, the primary indication for a heart-lung block was pulmonary arterial hypertension (PAH).^1^

Notably, the technical feasibility of a single and, later, bilateral lung transplantation was developed in Toronto, only several years later. The primary indications for lung-only transplantation back then were emphysema and fibrosis. Lung transplantation for PAH was not considered a viable option, primarily because the heart was considered too damaged due to chronic strain from prolonged elevated pulmonary pressures.

This view began to shift in the 1990s, when reports emerged demonstrating that lung-only transplantation was indeed feasible for PAH.^2^, ^3^, ^4^ The underlying rationale of a lung-only approach was the assumption that the heart, being a muscle could adapt – like other muscles – through training to accommodate changing physiological demands. This process is called reverse cardiac remodeling. Although single-lung transplantation has been performed for PAH,^5^ several studies have demonstrated superior outcomes with bilateral lung transplantation, including lower rates of primary graft dysfunction (PGD) and bronchiolitis obliterans syndrome, as well as improved overall survival.^6^, ^7^

By that time, bilateral lung transplantation has meanwhile become a highly standardized procedure with acceptable perioperative risks in other indications. The major advantage of a lung-only strategy was the growing awareness of organ availability issues and the persistent shortage of donor hearts.^8^, ^9^ Thus, a lung-only transplant strategy for PAH represents an organ sparing approach for treating patients with PAH.

Despite these developments, over the past two decades, significant concerns have continued to be raised regarding the lung-only approach, leading to the continued use of heart-lung transplantation for PAH in certain cases. Key concerns include right ventricular (RV) failure, left ventricular (LV) impairment, high rates of PGD, and challenges in managing PAH patients with associated structural defect of the heart (Figure 1).

**Figure 1:**
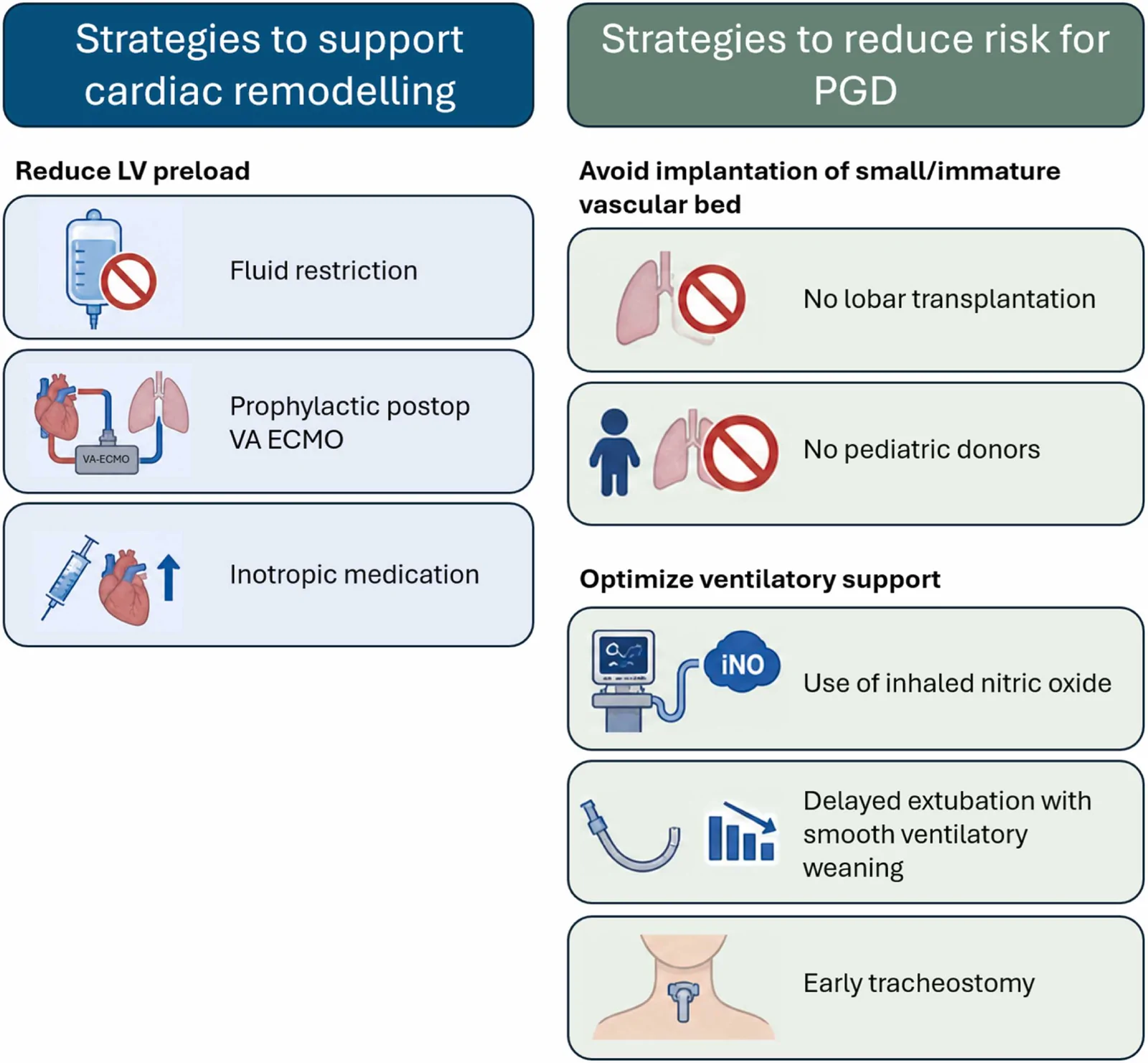
Strategies to support cardiac remodeling and protect the lung allograft in patients with pulmonary arterial hypertension undergoing lung transplantation.

## Pathophysiological considerations and patient management

**Right ventricle:** In the pre-transplant setting, the RV of a patient with pulmonary arterial hypertension chronically adapts to the increased afterload caused by the elevated vascular resistance. This leads to RV hypertrophy and dilation. In severe PAH, one of the most feared pre-transplant complications is therapy-refractory right ventricular failure, where the RV can no longer cope with the high vascular resistance – despite maximal medical treatment. However, this situation changes once donor lungs – characterized by normal vascular resistance – are implanted.^10^ In the vast majority of cases, the RV adapts well after lung transplantation. Nonetheless, in patients with severely hypertrophic RVs, functional outflow tract obstruction can occur.^11^ In this rare subgroup, careful management of RV afterload is crucial, i.e. avoid overly negative balancing. Even in cases with a pre-transplant RV failure, including those requiring pre-transplant ECMO support, outcomes following bilateral lung transplantation are acceptable – provided patients can be stabilized and proceed to transplant.^12^ This is supported by cMRI analysis evaluating right ventricular ejection fraction (RVEF) in patients with severely impaired RV function before transplant.^13^ Following transplantation, significant improvement in RV function has been observed. RVEF increased from 32% to 64% accompanied by a reduction in RV volume and mass. All this evidence – reflected in the most recent ISHLT consensus guidelines – that RV dysfunction alone does not necessitate heart-lung transplantation.^14^

**Left ventricle:** During the phase of reverse cardiac remodeling, the left ventricle must be carefully monitored. Prior to transplantation, the LV is typically adapted to low preload and low filling pressures. Following transplantation, however, the normalization of pulmonary vascular resistance causes an abrupt increase in preload, which can overwhelm the chronically untrained LV.^15^, ^16^ To mitigate this risk, many centers have adopted the strategy of prophylactic, prolonged veno-arterial extracorporeal membrane oxygenation (VA-ECMO) in patients with PAH. This approach reduces transpulmonary flow and prevents overloading the LV with volumes it is not accustomed to handle. VA-ECMO can usually be weaned after 2–5 days once patient’s fluid status has normalized.^17^, ^18^ The combination of VA-ECMO prolongation, aggressive diuresis, and early implementation of hemodiafiltration has been shown to be reduce the incidence of perioperative LV failure.^19^, ^20^ Cardiac MRI data indicate that the LV can fully recover its function after the remodeling period post-transplantation. In cMRI studies, there was also significant improvement in LV eccentricity index (2.8–1.1, p <0.001) and RV/LV volume ratio (2.3–0.8, p <0.001).^13^

**Structural defects of the heart:** With growing experience in managing the perioperative challenges of lung transplant recipients with PAH, some centers have begun offering lung-only transplants to patients with repairable structural heart defects. Based on surgical expertise, it is feasible to address minor structural defects of the heart during transplantation. Supporting case reports describe successful strategies such as concomitant or atrial septal defect (ASD) closures after lung transplantation,^21^, ^22^ repair of a pulmonary arterial (PA) trunk aneurysms (with or without PA valve repair),^23^, ^24^ and correction of partial anomalous pulmonary venous (PV) return.^25^ Notably, as catheter-based technologies advance, many of these minor structural cardiac abnormalities can now be addressed post-transplant through minimally invasive interventions. This has enabled some patients to undergo a lung-only transplant followed by catheter-based repair.^26^

Based on the data and experience outlined above, the indications for a heart-lung block in patients with PAH have become extremely rare. A heart-lung transplant should only be considered for PAH patients with severe concomitant structural heart defects – such as non-atrial Eisenmenger physiology – or for patients with grown-up congenital heart disease (GUCH) and fixed pulmonary hypertension. The latter group is particularly complex, and ongoing discussions continue regarding the optimal timing for heart-lung transplantation, as many of these patients can remain relatively stable and be managed conservatively for years.

## Outcomes

Direct comparison of the outcomes between heart-lung and lung-only transplantation currently favors lung-only approaches. In fact, median survival after lung-only transplantation now exceeds that of heart-lung transplantation in patients with PAH (Table 1).^27^, ^28^ There are several reasons for this trend. First, heart-lung transplantation is a more complex procedure, and the required expertise is becoming increasingly rare, with fewer than five centers worldwide performing more than four heart-lung transplants per year.^29^ As with other rare and technically demanding interventions, centralization may help ensure appropriate expertise and optimize outcomes. We therefore believe that HLTx should be concentrated in a small number of highly experienced centers, supported by structured nationwide referral pathways.

**Table 1:** Advantages and disadvantages of lung-only versus heart-lung transplantation for PAH. Table adapted from Huston et al. ^27^

	**Lung-only**	**Heart-lung**
**Pro**	Better donor organ allocation equity	Option for non-atrial Eisenmenger physiology and complex congenital heart disease
	Less complex surgical procedure	
**Cons**	Use of ECMO postoperatively	Longer wait list times
	Longer mechanical ventilation	Scarce resource use
		Dual organ rejection surveillance
**Median survival (years)**	7.1	6.5

Notably, with the advances in lung-only strategies for PAH, heart-lung transplantation is now typically reserved for complex cases, making recipients less comparable in terms of preoperative disease severity and post-transplant outcome. Additionally, heart-lung transplant recipients typically undergo more intensive rejection monitoring, requiring roughly twice the number of follow-up procedures, including imaging, pulmonary function tests, and both lung and heart biopsies. In many parts of the world, heart-lung candidates are disadvantaged compared those eligible for lung-only transplantation. For instance, in the U.S., current allocation policies result in longer wait times for combined heart-lung transplants than for lung-only procedures. This is significant, as transplant outcomes in PAH patients are strongly time dependent. Waitlist mortality is higher among PAH and increases substantially over time – with a 10% increase in 1-year mortality risk by day 30 on the waitlist.^30^

## Conclusions

Bilateral lung transplantation is considered the standard of care for patients with PAH requiring transplantation. Although perioperative management can be challenging, long-term outcomes are excellent. Heart-lung transplantation should be reserved for a carefully selected subgroup of patients in whom meaningful cardiac recovery after bilateral lung transplantation is unlikely and patients with major structural cardiac abnormalities.

## Disclosures

The authors report no conflicts of interest. Artificial intelligence (ChatGPT) was used for language editing and to generate the pictograms included in Figure 1.

## Funding

This work was supported by the Mrs. and Mr. Ragland Fund.

## Declaration of Competing Interest

The authors declare that they have no known competing financial interests or personal relationships that could have appeared to influence the work reported in this paper.
